# Age‐related differences in the immune response could contribute to determine the spectrum of severity of COVID‐19

**DOI:** 10.1002/iid3.404

**Published:** 2021-02-10

**Authors:** Giorgio Costagliola, Erika Spada, Rita Consolini

**Affiliations:** ^1^ Division of Pediatrics, Department of Clinical and Experimental Medicine, Section of Rheumatology and Clinical Immunology University of Pisa Pisa Italy

**Keywords:** ACE2, children, coronavirus, cytokine storm, immune dysregulation, multisystem inflammatory syndrome in children (MIS‐C)

## Abstract

Coronavirus disease 2019 (COVID‐19), can present with a wide spectrum of severity. Elderly patients with cardiac, pulmonary and metabolic comorbidities are more likely to develop the severe manifestations of COVID‐19, which are observed in less than 5% of the pediatric patients. Severe acute respiratory syndrome coronavirus 2 (SARS‐CoV‐2) is able to induce an immune impairment and dysregulation, finally resulting in the massive release of inflammatory mediators, strongly contributing to the pulmonary and systemic manifestations in COVID‐19. In children, the immune dysregulation following SARS‐CoV‐2 can also be responsible of a severe disease phenotype defined as multisystem inflammatory syndrome in children. As the immune system undergoes a complex process of maturation from birth to adult age, differences in the immune and inflammatory response could have a significant impact in determining the spectrum of severity of COVID‐19. Indeed, children show a higher ability to respond to viral infections and a reduced baseline pro‐inflammatory state compared with elderly patients. Age and comorbidities contribute to disease severity through immune‐mediated mechanisms, since they are associated with a chronic increase of pro‐inflammatory mediators, and cause an enhanced susceptibility to develop an immune dysregulation following SARS‐CoV‐2 infection. Also the expression of ACE2, the receptor of SARS‐CoV‐2, varies with age, and is linked to the immune and inflammatory response through a complex, and not completely elucidated, network. This paper reviews the peculiar immunopathogenic aspects of COVID‐19, with a focus on the differences between adult and pediatric patients.

## Graphical abstract

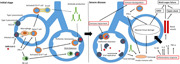

## 1 INTRODUCTION

Coronavirus disease 2019 (COVID‐19), caused by severe acute respiratory syndrome coronavirus 2 (SARS‐CoV‐2) infection, is featured by a variable spectrum of severities. Although a significant percentage of patients present with upper respiratory infection, COVID‐19 can be associated with the development of pneumonia and, in severe cases, acute respiratory distress syndrome (ARDS), septic shock, disseminated intravascular coagulation, and multiorgan failure (MOF).^1^ The pathogenesis of COVID‐19 has not been completely elucidated. Nevertheless, it is widely recognized that virus‐related factors are involved in the complex pathogenic mechanism of the disease, with the immune system playing a central role in the development of the severe disease. Indeed, during infection, an aberrant immune response can be elicited, resulting in the massive release of cytokines and chemokines (“cytokine storm”), which cause pulmonary and systemic tissue damage, leading to the clinical manifestations of severe COVID‐19.^2^ Therefore, different drugs active on the immune system, including anti‐cytokine agents have been proposed for the prevention and treatment of severe COVID‐19.

Since the first description of COVID‐19, it clearly emerged that disease severity appears to be higher among elderly patients with significant comorbidities (chronic cardiac or pulmonary disorders, obesity, and diabetes)^3^, ^4^ while pediatric patients frequently have a mild clinical course.^5^, ^6^, ^7^, ^8^ Indeed, about 20% of the adult patients develop the severe or critical manifestations of COVID‐19, requiring admission to an intensive care unit (ICU),^1^ while the admission rate to ICU is 2%–3% in children, in which the fatality rate is lower than 0.1%.^5^, ^9^ Interestingly, a study by Parri et al.^8^ evidenced that, among children, those ones under six months of age show a higher risk of developing severe or critical disease.^9^

Beyond disease severity, also differences in the clinical phenotype of COVID‐19 between adult and pediatric patients have been observed. In particular, although even in pediatric age the most commonly reported clinical findings are fever and signs of respiratory infection (dry cough, myalgia, rhinitis, sore throat, and less frequently, pneumonia),^10^, ^11^, ^12^ gastrointestinal symptoms, such as vomiting, abdominal pain, and diarrhea, are more common in children compared with the adult population.^13^

Given the rarity of severe COVID‐19 in children and adolescent, the knowledge of the mechanisms underlying its pathogenesis is still limited. However, peculiarities of the immune and inflammatory response in pediatric age, together with age‐related extra‐immunological factors, such as Angiotensin I Converting Enzyme 2 (ACE2) receptor expression and comorbidities, could significantly contribute in determining the different clinical phenotype and disease severity between adult and pediatric patients. This paper reviews the main immunopathogenic aspects of COVID‐19, with a focus on the age‐related differences between adult and pediatric patients and the deriving clinical implications.

## 2 IMMUNE PATHOGENESIS OF COVID‐19: AN OVERVIEW

The interplay between the effectiveness of the immune response to allow viral clearance and the elevated inflammatory response that determines lung damage is crucial for both the understanding of the disease course and the identification of potential therapeutic targets. Therefore, COVID‐19 is at an intriguing “crossroad” between an infectious disease and an immune/autoinflammatory disorder.

### 2.1 The immune response

Both the innate and adaptive immune systems are involved in the physiological response to coronaviruses (CoVs). After the ACE2‐mediated entry into the cells, virus‐associated patterns are recognized by toll‐like receptors (TLR), expressed by the cells of the innate immune system, and in particular TLR‐7.^14^ The activation of TLR is followed by the initiation of several signaling pathways, leading to the production of pro‐inflammatory cytokines, including Type 1 and Type 3 interferon (IFN‐1, IFN‐III).^15^, ^16^ IFN‐1, through the activation of Janus kinase–signal transducer and activator of transcription (JAK/STAT) signaling,^17^ promotes its antiviral effects, enhancing phagocytosis, chemotaxis and, finally, the clearance of the infectious agent mediated by natural killer (NK) cells and the activation of the adaptive response.^15^

Following phagocytosis, the viral proteins are internalized in the macrophages, and then presented on Class‐I and Class‐II major histocompatibility complex molecules, to activate the adaptive immune response. In the context of the adaptive response to CoVs, T‐cells, particularly the T CD8+ subset, play a major role in the clearance of the infectious agents.^18^ Concerning humoral immunity, the role of antibodies seems prominent in the persistent phase of the infection by COVs.^15^ In the specific case of COVID‐19, the timing of seroconversion is reported to be a median of 11–14 days,^19^ but currently it is not possible to determine the duration of a protective antibody titer.

### 2.2 The host immune impairment

CoVs have molecular mechanisms that allow escape from the host immune response. In SARS‐CoV and MERS‐CoV infection, authors observed the ability of the virus to interfere, through both TLR‐dependent and independent pathways, with the production of IFN‐1 and IFN‐III and the appropriate initiation of the immune response.^15^, ^20^ This action depends on different mechanisms: impairment of the pathogen recognition, reduced cellular IFN production (through the inhibition of the IRF3 transcription factor), and altered IFN‐mediated signaling, finally reducing both the expression and function of the IFN‐stimulated genes (ISGs).^16^ High levels of IFN and ISGs are evidenced in patients with complicated disease course in severe acute respiratory syndrome (SARS)^21^ and Middle East respiratory syndrome (MERS),^22^ suggesting that severe infections could trigger IFN response, which is, however, ineffective in reducing the viral load.^16^ Moreover, in patients with COVID‐19, and particularly in severe forms of the disease, it is possible to observe alterations in the lymphocyte count and in specific lymphocyte subsets,^23^ suggesting a potential interference of the virus with adaptive immunity.

Patients with COVID‐19 have a reduction of lymphocyte count, T CD4+ and T CD8+ subpopulations, and absolute NK cell count compared with standard reference values. Interestingly, patients with severe disease show a more pronounced impairment in the adaptive immune response. In particular, they more frequently show lymphocytopenia,^24^ and the absolute values of T CD4+ and T CD8+ cells are significantly reduced compared with patients with a mild disease course.^23^, ^25^ Unlike other CoVs, Zheng et al.^26^ reported a reduced functional diversity of T CD4+ cells together with an elevation of T CD8+ exhausted cells in the peripheral blood of patients with a severe disease course, which contributes to the ineffective immune response against SARS‐CoV‐2. A significant reduction of the T‐regulatory cell subset has also been described, contributing to infection‐related immune dysregulation.^23^ An impaired immune response could result in an enhanced replication of the infectious agent, with consequent organ damage and further activation of the inflammatory process, responsible for the subsequent clinical worsening of the disease.

### 2.3 The host immune dysregulation: A cytokine storm beyond the infectious disease

Several studies have reported that patients with severe disease have markedly elevated serum levels of several pro‐inflammatory cytokines, including interleukin‐6 (IL‐6), IL‐1, IFN‐γ, and tumor necrosis factor‐α (TNF‐α). A significant elevation of other mediators associated with inflammation, particularly ferritin and C‐reactive protein (CRP), is also observed in this disease stage. By analyzing a large cohort of COVID‐19 patients, Quin et al.^23^ showed that the elevation of IL‐6, IL‐8, IL‐10, CRP, and ferritin in patients with severe disease was statistically significant compared with mild disease. In the pathogenesis of COVID‐associated hyperinflammation, the role of IL‐6 is crucial,^27^ as the cytokine is able both to enhance the inflammatory response by polymorphonucleate cell activation (phase of local inflammatory response)^28^ and to maintain it by macrophage activation and amplification of the response to TLR activation^29^ (phase of prolonged inflammation).

**Figure 1:**
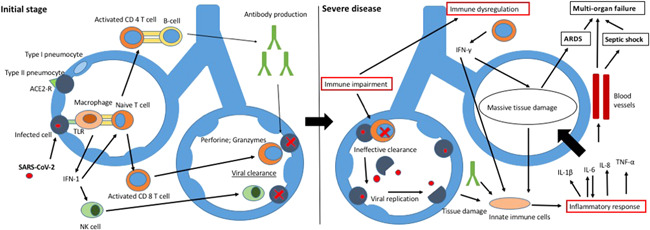
Pathogenic model of severe COVID‐19. The left side of the figure shows the activation of innate and adaptive immune response following viral infection. The right side analyzes how impaired immunity, immune dysregulation, and uncontrolled inflammation led to massive tissue damage, thus enhancing the inflammatory response, finally leading to ARDS, septic shock and MOF. ARDS, acute respiratory distress syndrome; MOF, multiorgan failure

Taken together, the evidence derived from analysis of the immune response to SARS‐CoV‐2, the immune‐escaping mechanisms, the host immune impairment, and the cytokine profile seen in patients with a severe course, supports the hypothesis of a two‐phase immune response in COV infection, particularly in the severe COVID‐19 form. In the first stage, the host elicits the development of an endogenous adaptive immune response to eliminate the virus and to preclude disease progression. If the protective specific immunity is impaired, the host enters the second phase, characterized by virus propagation and an uncontrolled inflammatory response leading to massive organ damage (Figure 1).

The cytokine profile described in severe patients with COVID‐19 shows common features with that detected in patients with hemophagocytic lymphohistiocytosis (HLH) macrophage activation syndrome (MAS), developing in patients with rheumatologic diseases and cytokine release syndrome (CRS), an adverse effect following the administration of chimeric antigen receptor‐T cells (CAR‐T‐cells) in the treatment of several malignancies. In patients with HLH–MAS, a significant increase of several pro‐inflammatory cytokines, including IL‐1β, IL‐6, IL‐18, TNF‐α (produced by monocyte‐macrophages), IL‐2, and IFN‐γ, of lymphocytic origin, together with hyperferritinemia, cytopenias (mainly thrombocytopenia), and impaired liver function is observed. Clinically, these patients experience unremitting fever, hepatic dysfunction, hepatosplenomegaly, and potential progression to MOF.^30^, ^31^ Although the first‐line treatment of MAS mostly relies on corticosteroids and cyclosporine and data on the use of cytokine blockade strategies, are controversial,^32^ the role of biologic agents (including anti‐IL‐1 and anti‐IL‐6 drugs) in the therapeutic approach to MAS is still being investigated in clinical trils.^32^, ^33^ In CRS a similar clinical picture with multysistemic involvement is observed, with the pathogenesis deriving from the massive release of cytokines derived from the expanded CAR‐T‐cells (IFN‐γ and IL‐2) and from activated cells from innate immunity (IL‐6, IL‐8, IL‐10, and others).^34^ In the treatment of CRS, the main option is the use of the anti‐IL‐6 monoclonal antibody tocilizumab,^34^ approved in both adults and children. These similarities between COVID‐19 and the above described cytokine storm syndromes support the rationale use of anti‐cytokine agents in the treatment of severe COVID‐19.^35^

## 3 PECULIAR IMMUNE PATHOGENIC ASPECTS OF CHILDHOOD‐ONSET DISEASE

The question regarding why children are less susceptible to COVID‐19 remains unresolved. As we assume that host immune responsiveness plays a major role in determining both the clinical phenotype and the disease course, we are focusing on the differences in the host immune response to COVID‐19 between childhood and adulthood (Table 1). It should be noted that the immune system undergoes a complex process of maturation, beginning during the fetal stage and culminating in adolescence, with progressive acquisition of a complete immune function. Furthermore, aging is accompanied by remodeling of the immune system; with time, this leads to a decline in immune efficacy, resulting in increased vulnerability to infectious diseases and a susceptibility to age‐related inflammatory disease. This potentially explains the different severity of COVID‐19 in elderly patients.

**Table 1:** Peculiarity in the immune response in pediatric age with relevance in COVID‐19

Cellular adaptive immunity	Humoral adaptive immunity
↑ Thymic output	↑ Bone marrow output
↑ Repertoire of naive T‐cells	↑ Functional capacity of B‐cells
↑ Absolute values of CD4+ cells	**Innate immunity and inflammation**
↑ Absolute values of CD8+ cells	↑ Baseline tone of innate immunity
↑ Expression of CD27, CD28	↑Levels of mDC and pDC
	↓ Baseline pro‐inflammatory state

Abbreviations: COVID‐19, coronavirus disease 2019; mDC, myeloid dendritic cells; pDC, plasmacytoid dendritic cells.

### 3.1 The host immune responsiveness in children

Children have a higher proportion of naive T‐cells than adults, and therefore a more pronounced ability both to respond to new pathogens and to eliminate infectious agents.^14^ With aging, continuous antigen stimulation and thymic involution leads both to a decrease in peripheral T‐cell reservoirs and to a shift in the T‐cell subset distribution from naive T‐cells to memory T‐cells.^36^ Naive CD4+ and CD8+ T‐cells decrease linearly with age; CD8+ twice more rapidly. Memory cells outnumber naive cells on average at 37.4 years in the CD4+ and 29.5 years in the CD8+pool, which could indicate an acceleration of the decay of the immune system from this age onward.^37^ Based on changes in memory T‐cell frequency, pathogen susceptibility, and mortality throughout human life, Farber et al.^38^ divided an individual's lifetime into three phases: memory generation (ages 0–20 years), memory homeostasis (ages 20–65 years), and immunosenescence (age > 65 years). This process is also accompanied by the loss of expression of costimulatory molecules, such as CD27 and CD28. CD28, an important T‐cell costimulatory receptor, is responsible for T‐cell activation, proliferation, and survival.^37^, ^39^ Moreover, the accumulation of CD28‐T‐cells mainly contributes to age‐associated changes in T‐cells, leading to a reduced overall immune response to pathogens and vaccines.^39^, ^40^ Studies on children with severe COVID‐19 showed a decrease in the NK subpopulation together with an increase in T CD4 and CD8 subpopulation,^41^ differently from adult patients. Interestingly, pediatric patients are less likely to develop lymphocytopenia.^5^, ^42^

With regard to humoral immunity, early descriptive studies of age‐associated changes in the B‐cell lineage revealed diminished bone marrow output and a reduction in the functional capacities of B‐cells and their progenitors, with changes in the sizes of different subsets and shifts in the diversity and clonotypic composition of the antigen‐responsive repertoire.^40^, ^43^ Interestingly, the accumulation of a subset of atypical B‐cells, termed age‐associated B‐cells (ABCs), is one of the key age‐related changes in B‐cell compartment. ABCs, with their inherent capacity for secreting antibodies, cytokines, and presenting antigens, may play an important role in the complicated signaling network associated with immune senescence.^44^

Finally, also age‐related differences in the innate immune response can influence the clinical course of SARS‐CoV‐2 infection. Peripheral dendritic cells (DCs; both myeloid and plasmacytoid subsets) show a significant decline with age. As DCs are the most important antigen‐presenting cells, playing a pivotal role in T‐cell function and in the link between innate and adaptive immunity, their decline through life is a relevant contributor to the increased susceptibility to infections with age.^45^ It is important to note that the plasmacytoid subset produces very high levels of Type I IFN upon stimulation with viruses, playing a central role in antiviral immunity. Recent observations suggested that trained innate immunity could contribute to the reduced severity of COVID‐19 in pediatric age.^46^ Indeed, the exposure to microbial antigens, vaccines and adjuvants can increase the baseline reactivity of the innate immune system against infectious agents, through functional and epigenetic modifications in progenitors of the myeloid lineage in the bone marrow.^47^ Children receive, in the first years of life, a high number of vaccines^48^ (frequently containing adjuvants) and experience a significant number of respiratory infections. Therefore, it can be hypothesized that children could have a more pronounced “tone” of the innate immunity compared to adult patients, contributing both in the protection against SARS‐CoV‐2 infection and in the mitigation of the clinical course in the infected patients.^46^

### 3.2 The immune dysregulation in children

The “cytokine storm” has been demonstrated in pediatric patients with severe disease, with a detectable elevation of IL‐6, IFN‐γ, and IL‐10. Interestingly, elevated levels of IL‐6 and IL‐10 are associated with longer disease duration and the need for intensive care support.^41^ Given the high levels of activated T‐cells evidenced in severe pediatric COVID‐19, a theory is that the cytokine storm in pediatric patients could be dependent both on a primary response to massive cell death and on a secondary, T‐cell‐dependent, immune response, which enhances the cytokine release.

The dysregulation of the inflammatory response has a particular clinical relevance in pediatric population. Indeed, in children the infection by SARS‐CoV‐2 can lead to the development of a multysistemic inflammatory syndrome in children (MIS‐C), a potentially life‐threatening condition which is often featured by a Kawasaki‐like clinical picture.^49^, ^50^, ^51^ In most of the cases, children with MIS‐C show positive serology for SARS‐CoV‐2, while the positivity of the nasopharyngeal real‐time polymerase chain reaction is reported in less than 40% of the affected children.^52^ The pathogenic mechanism leading to MIS‐C is not completely elucidated, and different theories have been formulated, investigating the role of both innate and adaptive immune response. With regard to innate immunity, the available evidence suggest that a delayed IFN response, caused by the previously described ability of CoVs to impair IFN‐I and IFN‐III synthesis and action,^16^ could lead to a slower viral clearance, and be accompanied by an enhanced systemic inflammatory response, with high levels of different cytokines, including IL‐1 and IL‐6.^35^, ^53^ On the other hand, the delay between SARS‐CoV‐2 infection and the development of MIS‐C (approximately 4–6 weeks), together with the clinical efficacy of intravenous immunoglobulins in its treatment, suggest the involvement of the adaptive immune system in the pathogenesis of this condition. A study by Consiglio et al.^54^ evidenced that children with MIS‐C have low levels of T CD4+ cells and an increase of the senescent T cell population, suggesting that a defective T cell response could participate in the pathogenesis of MIS‐C. In the same study, from the analysis of the autoantibody profile emerged different candidates for a pathogenic role in MIS‐C, although definitive associations were not identified.^54^

## 4 AGE‐RELATED ROLE OF EXTRA‐IMMUNOLOGICAL FACTORS IN THE PATHOGENESIS OF COVID‐19

It is noteworthy that immune‐related factors are not the only determinants of the different disease severity in the adult population. As previously discussed, patients with COVID‐19 suffering from chronic cardiac disease, diabetes, chronic pulmonary disorders, hypertension, and renal disorders present more frequently with a severe disease course. Although the most accepted explanation for this increased severity is represented by the reduced baseline cardiac and respiratory function, age and comorbidities can influence the outcome after SARS‐CoV‐2 infection also through immune‐and inflammatory‐mediated mechanisms. Indeed, aged patients with comorbidities, chronic vascular and microvascular injury (i.e., related to hypertension), and smoking habits, have a more pronounced baseline pro‐inflammatory state in the lungs, which contributes to the development of immune dysregulation observed in COVID‐19.^55^ Age itself is associated with an increase in the systemic and tissue levels of different pro‐inflammatory mediators and reactive species of oxygen, in realizing a condition defined as “inflame‐aging.”^56^ Moreover, as previously discussed, the ineffective immune response in aged patients is associated with impaired viral clearance,^57^, ^58^ enhancing the availability of viral antigens and primary tissue damage. Consequently, it is reasonable to postulate that aged patients are more prone to develop an uncontrolled immune response after virus‐induced tissue injury,^59^ contributing to the higher severity of COVID‐19 in adult ages. Similarly, the higher rate of severe COVID‐19 in patients with obesity^4^ can partly depend on a systemic inflammatory state. Indeed, in obese patients the localized inflammatory reaction in the adipose tissue, associated with the production of leptin and the reduced levels of adiponectin, can lead to a chronic low degree systemic inflammation, featured by the activation of cells of the innate ad adaptive immunity (Th1, and T CD8+ lymphocytes) and by the evidence of increased levels of different pro‐inflammatory cytokines, including IL‐6 and TNF‐α.^60^, ^61^ The upregulated inflammatory response in obese patients, together with the endothelial dysfunction and the increased expression of ACE2 associated with obesity,^62^ are important factors in determining the increased severity of COVID‐19 in this population.^63^

### 4.1 Age‐related influence on ACE2 receptor

Since the first description of the central role of ACE2 receptor in the infectious cycle of SARS‐CoV‐2, several studies focused on the age‐related differences in the expression of the ACE2 receptor. Although a progressive decrease of the receptor expression with age has been described,^64^ other authors reported a lower density of ACE2 in children compared to adult patients.^65^ Most of the studies reported that the expression of ACE2 is enhanced in smokers and obese patients,^62^ while data on the tissue levels of ACE2 in systemic and pulmonary disorders are not univocal.^66^, ^67^, ^68^, ^69^, ^70^ However, the interest on ACE2 receptor in COVID‐19 is not limited to its role of binding site for SARS‐CoV‐2. Indeed, ACE2 is involved in the viral replication, in the activation of the immune and inflammatory response (with secretion of pro‐inflammatory cytokines) and participates to the delicate equilibrium regulating the development of pulmonary damage during the infection. With regard to the involvement of ACE2 in other phases of the infectious process, recent works suggests that ACE2 could enhance the expression of genes involved in viral replication.^66^ Moreover, a study by Ziegler et al.,^71^ evidenced that ACE2 expression is stimulated by IFN, suggesting that SARS‐CoV‐2 could up‐regulate ACE‐2 expression in an IFN‐mediated mechanism, thus enhancing the infectious process. Also the effects of ACE 2 on the immune system during CoVs infections have been extensively analyzed. Studies on SARS‐CoV infection demonstrated that the expression of ACE2 was associated with the tissue secretion of the pro‐inflammatory cytokines involved in the pathogenesis of ARDS, and that its expression was correlated with disease severity.^72^ This finding was evidenced also in COVID‐19 in a recent study by Li et al.,^66^ which demonstrated an association between ACE2 expression and the levels of different pro‐inflammatory cytokines and cellular subpopulations (Th1 and Th17 cells) involved in the adaptive response. Finally, it is important to underline that, as demonstrated in experimental studies on models of pulmonary damage caused by respiratory syncytial virus and by the intraperitoneal administration of bleomycin,^73^, ^74^ ACE2 has a protective role against the development of lung injury. ACE2 acts by inhibiting the apoptosis of pulmonary endothelial cells during the acute phase of lung injury through multiple mechanisms, including the upregulation of Bcl‐2 protein and the suppression of Mir‐4262.^74^ However, the complex interactions between ACE2 receptor, SARS‐CoV‐2, the immune system and pulmonary tissue are still undefined. Similarly, age‐related differences in the expression, function and maturity of ACE2 and the mechanisms by which this protein influences the clinical phenotype of the disease deserve further investigation.

## 5 CONCLUSION

The immune and inflammatory responses play a significant role in controlling SARS‐CoV‐2 infection, but can also be concurring factors in the pathogenesis of the pulmonary and systemic manifestations of severe COVID‐19. Age‐related variations in the number and function of cells of the innate and adaptive immune response, together with the complex interactions between ageing, comorbidities and the immune and inflammatory response could contribute to determining the higher disease severity in the elderly patients and the milder disease course frequently observed in children. As drugs targeting the immune response are used in both adult and pediatric patients to prevent and treat the severe disease, we hope that a better characterization of the age‐related differences in the pathogenesis of COVID‐19, could open the opportunity to critically improve the therapeutic approach to the disease in both populations.

## CONFLICT OF INTERESTS

The authors declare no conflict of interests.

## AUTHOR CONTRIBUTIONS

Giorgio Costagliola and Erika Spada wrote the manuscript, which was critically revised by Rita Consolini. All authors contributed to manuscript revisions, and read and approved the submitted version.

## Data Availability

Data sharing is not applicable to this article as no new data were created or analyzed in this study.

## References

[1] Rodriguez‐Morales AJ , Cardona‐Ospina JA , Gutiérrez‐Ocampo E , et al. Clinical, laboratory and imaging features of COVID‐19: a systematic review and meta‐analysis. Travel Med Infect Dis. 2020;34:101623. 32179124 10.1016/j.tmaid.2020.101623 PMC7102608

[2] Sarzi‐Puttini P , Giorgi V , Sirotti S , et al. COVID‐19, cytokines and immunosuppression: what can we learn from severe acute respiratory syndrome? Clin Exp Rheumatol. 2020;38(2):337‐342. 32202240

[3] Zheng Z , Peng F , Xu B , et al. Risk factors of critical & mortal COVID‐19 cases: a systematic literature review and meta‐analysis. J Infect. 2020;81(2):e16‐e25. 10.1016/j.jinf.2020.04.021 PMC7177098 32335169

[4] Yang J , Hu J , Zhu C . Obesity aggravates COVID‐19: a systematic review and meta‐analysis. J Med Virol. 2020:jmv.26677 10.1002/jmv.26237 PMC7361606 32603481

[5] Liguoro I , Pilotto C , Bonanni M , et al. SARS‐COV‐2 infection in children and newborns: a systematic review. Eur J Pediatr. 2020;179(7):1029‐1046. 32424745 10.1007/s00431-020-03684-7 PMC7234446

[6] Dong Y , Mo X , Hu Y , et al. Epidemiological characteristics of 2143 pediatric patients with 2019 coronavirus disease in China. Pediatrics. 2020.

[7] CDC COVID‐19 Response Team . Coronavirus Disease in Children ‐ United States, February 12‐April 2, 2020. MMWR Morb Mortal Wkly Rep. 2019;69(14):422‐426. 10.15585/mmwr.mm6914e4 PMC7147903 32271728

[8] Parri N , Magistà AM , Marchetti F , et al. Characteristic of COVID‐19 infection in pediatric patients: early findings from two Italian Pediatric Research Networks. Eur J Pediatr. 2020;179(8):1315‐1323. 32495147 10.1007/s00431-020-03683-8 PMC7269687

[9] Zimmermann P , Curtis N . COVID‐19 in children, pregnancy and neonates: a review of epidemiologic and clinical features. Pediatr Infect Dis J. 2020;39(6):469‐477. 32398569 10.1097/INF.0000000000002700 PMC7363381

[10] Mantovani A , Rinaldi E , Zusi C , Beatrice G , Saccomani MD , Dalbeni A . Coronavirus disease 2019 (COVID‐19) in children and/or adolescents: a meta‐analysis. Pediatr Res. 2020. 10.1038/s41390-020-1015-2 32555539

[11] de Souza TH , Nadal JA , Nogueira RJN , Pereira RM , Brandão MB . Clinical manifestations of children with COVID‐19: A systematic review. Pediatr Pulmonol. 2020;55(8):1892‐1899. 32492251 10.1002/ppul.24885 PMC7300659

[12] She J , Liu L , Liu W . COVID‐19 epidemic: disease characteristics in children. J Med Virol. 2020;92:747‐754. 32232980 10.1002/jmv.25807 PMC7228385

[13] Zhou MY , Xie XL , Peng YG , et al. From SARS to COVID‐19: what we have learned about children infected with COVID‐19. Int J Infect Dis. 2020;96:710‐714. 32389849 10.1016/j.ijid.2020.04.090 PMC7204709

[14] Ahmadpoor P , Rostaing L . Why the immune system fails to mount an adaptive immune response to a COVID‐19 infection. Transpl Int. 2020;33:824‐825. 32236983 10.1111/tri.13611

[15] Li G , Fan Y , Lai Y , et al. Coronavirus infections and immune responses. J Med Virol. 2020;92(4):424‐432. 31981224 10.1002/jmv.25685 PMC7166547

[16] Park A , Iwasaki A . Type I and Type III interferons ‐ induction, signaling, evasion, and application to combat COVID‐19. Cell Host Microbe. 2020;27(6):870‐878. 32464097 10.1016/j.chom.2020.05.008 PMC7255347

[17] Ivashkiv LB , Donlin LT . Regulation of type I interferon responses. Nat Rev Immunol. 2014;14(1):36‐49. 24362405 10.1038/nri3581 PMC4084561

[18] Zhao J , Zhao J , Perlman S . T cell responses are required for protection from clinical disease and for virus clearance in severe acute respiratory syndrome coronavirus‐infected mice. J Virol. 2010;84(18):9318‐9325. 20610717 10.1128/JVI.01049-10 PMC2937604

[19] Zhao J , Yuan Q , Wang H , et al. Antibody responses to SARS‐CoV‐2 in patients of novel coronavirus disease 2019. Clin Infect Dis. 2020. 10.1093/cid/ciaa344 PMC7184337 32221519

[20] Lu X , Pan J , Tao J , Guo D . SARS‐CoV nucleocapsid protein antagonizes IFN‐beta response by targeting initial step of IFN‐beta induction pathway, and its C‐terminal region is critical for the antagonism. Virus Genes. 2011;42(1):37‐45. 20976535 10.1007/s11262-010-0544-x PMC7088804

[21] Cameron MJ , Ran L , Xu L , et al. Interferon‐mediated immunopathological events are associated with atypical innate and adaptive immune responses in patients with severe acute respiratory syndrome. J Virol. 2007;81(16):8692‐8706. 17537853 10.1128/JVI.00527-07 PMC1951379

[22] Kim ES , Choe PG . Clinical progression and cytokine profiles of Middle East Respiratory Syndrome Coronavirus. Infection. 2016;31(11):1717‐1725. 10.3346/jkms.2016.31.11.1717 PMC5056202 27709848

[23] Qin C , Zhou L , Hu Z , et al. Dysregulation of immune response in patients with COVID‐19 in Wuhan, China. Clin Infect Dis. 2020. 10.1093/cid/ciaa248 PMC7108125 32161940

[24] Yang X , Yu Y , Xu J , et al. Clinical course and outcomes of critically ill patients with SARS‐CoV‐2 pneumonia in Wuhan, China: a single‐centered, retrospective, observational study. Lancet. Respir Med. 2020;8:475‐481. 10.1016/S2213-2600(20)30079-5 PMC7102538 32105632

[25] Chen G , Wu D , Guo W , et al. Clinical and immunologic features in severe and moderate Coronavirus Disease 2019. J Clin Invest. 2020;130:2620‐2629. 32217835 10.1172/JCI137244 PMC7190990

[26] Zheng HY , Zhang M , Yang CX , et al. Elevated exhaustion levels and reduced functional diversity of T cells in peripheral blood may predict severe progression in COVID‐19 patients. Cell Mol Immunol. 2020;17:541‐543. 32203186 10.1038/s41423-020-0401-3 PMC7091621

[27] McGonagle D , Sharif K , O'Regan A , Bridgewood C . The role of cytokines including interleukin‐6 in COVID‐19 induced pneumonia and macrophage activation syndrome‐like disease. Autoimmun Rev. 2020;19:102537. 32251717 10.1016/j.autrev.2020.102537 PMC7195002

[28] Zhang C , Wu Z , Li JW , Zhao H , Wang GQ . The cytokine release syndrome (CRS) of severe COVID‐19 and Interleukin‐6 receptor (IL‐6R) antagonist Tocilizumab may be the key to reduce the mortality. Int J Antimicrob Agents. 2020;55:105954. 32234467 10.1016/j.ijantimicag.2020.105954 PMC7118634

[29] Strippoli R , Carvello F , Scianaro R , et al. Amplification of the response to Toll‐like receptor ligands by prolonged exposure to interleukin‐6 in mice: implication for the pathogenesis of macrophage activation syndrome. Arthritis Rheum. 2012;64(5):1680‐1688. 22108837 10.1002/art.33496

[30] Ravelli A , Minoia F , Davì S , et al. Classification criteria for macrophage activation syndrome complicating systemic juvenile idiopathic arthritis: a European League Against Rheumatism/American College of Rheumatology/Paediatric Rheumatology International Trials Organisation Collaborative Initiative. Ann Rheum Dis. 2016;75(3):481‐489. 26865703 10.1136/annrheumdis-2015-208982

[31] Schulert GS , Grom AA . Macrophage activation syndrome and cytokine‐directed therapies. Best Pract Res Clin Rheumatol. 2014;28(2):277‐292. 24974063 10.1016/j.berh.2014.03.002 PMC4074772

[32] Grom AA , Horne A , De , Benedetti F . Macrophage activation syndrome in the era of biologic therapy. Nat Rev Rheumatol. 2016;12(5):259‐268. 27009539 10.1038/nrrheum.2015.179 PMC5851441

[33] Weaver LK , Behrens EM . Weathering the storm: improving therapeutic interventions for cytokine storm syndromes by targeting disease pathogenesis. Curr Treatm Opt Rheumatol. 2017;3(1):33‐48. 28944163 10.1007/s40674-017-0059-x PMC5606329

[34] Thakar MS , Kearl TJ , Malarkannan S . Controlling cytokine release syndrome to harness the full potential of CAR‐based cellular therapy. Front Oncol. 2019;9:1529. 32076597 10.3389/fonc.2019.01529 PMC7006459

[35] Blanco‐Melo D , Nilsson‐Payant BE , Liu WC , et al. Imbalanced host response to SARS‐CoV‐2 drives development of COVID‐19. Cell. 2020;181(5):1036‐1045. 32416070 10.1016/j.cell.2020.04.026 PMC7227586

[36] Li M , Yao D , Zeng X , et al. Age related human T cell subset evolution and senescence. Immun Ageing. 2019;16:24. 31528179 10.1186/s12979-019-0165-8 PMC6739976

[37] Saule P , Trauet J , Dutriez V , Lekeux V , Dessaint JP , Labalette M . Accumulation of memory T cells from childhood to old age: central and effector memory cells in CD4( + ) versus effector memory and terminally differentiated memory cells in CD8( + ) compartment. Mech Ageing Dev. 2006;127(3):274‐281. 16352331 10.1016/j.mad.2005.11.001

[38] Farber DL , Yudanin NA , Restifo NP . Human memory T cells: generation, compartmentalization and homeostasis. Nat Rev Immunol. 2014;14(1):24‐35. 24336101 10.1038/nri3567 PMC4032067

[39] Weng NP , Akbar AN , Goronzy J . CD28(‐) T cells: their role in the age‐associated decline of immune function. Trends Immunol. 2009;30(7):306‐312. 19540809 10.1016/j.it.2009.03.013 PMC2801888

[40] Nicoletti C , Yang X , Cerny J . Repertoire diversity of antibody response to bacterial antigens in aged mice. III. Phosphorylcholine antibody from young and aged mice differ in structure and protective activity against infection with *Streptococcus pneumoniae* . J Immunol. 1993;150(2):543‐549. 8419487

[41] Sun D , Li H , Lu XX , et al. Clinical features of severe pediatric patients with coronavirus disease 2019 in Wuhan: a single center's observational study. World J Pediatr. 2020;16:251‐259. 32193831 10.1007/s12519-020-00354-4 PMC7091225

[42] Lu X , Zhang L , Du H , et al. SARS‐CoV‐2 infection in children. N Engl J Med. 2020;382:1663‐1665. 32187458 10.1056/NEJMc2005073 PMC7121177

[43] Cancro MP , Hao Y , Scholz JL , et al. B cells and aging: molecules and mechanisms. Trends Immunol. 2009;30(7):313‐318. 19540810 10.1016/j.it.2009.04.005 PMC2766868

[44] Ma S , Wang C , Mao X , Hao Y . B cell dysfunction associated with aging and autoimmune diseases. Front Immunol. 2019;10:318. 30873171 10.3389/fimmu.2019.00318 PMC6400972

[45] Orsini G , Legitimo A , Failli A , Massei F , Biver P , Consolini R . Enumeration of human peripheral blood dendritic cells throughout the life. Int Immunol. 2012;24(6):347‐356. 22345276 10.1093/intimm/dxs006

[46] Mantovani A , Netea MG . Trained innate immunity, epigenetics, and COVID‐19. N Engl J Med. 2020;383(11):1078‐1080. 32905684 10.1056/NEJMcibr2011679

[47] Laval B , Maurizio J , Kandalla PK , et al. C/EBPβ‐dependent epigenetic memory induces trained immunity in hematopoietic stem cells. Cell Stem Cell. 2020;26(5):657‐674. 32169166 10.1016/j.stem.2020.01.017

[48] MacDonald NE , Harmon S , Dube E , et al. Mandatory infant & childhood immunization: rationales, issues and knowledge gaps. Vaccine. 2018;36(39):5811‐5818. 30143274 10.1016/j.vaccine.2018.08.042

[49] Feldstein LR , Rose EB , Horwitz SM , et al. Multisystem inflammatory syndrome in U.S. children and adolescents. N Engl J Med. 2020;383(4):334‐346. 32598831 10.1056/NEJMoa2021680 PMC7346765

[50] Riphagen S , Gomez X , Gonzalez‐Martinez C , Wilkinson N , Theocharis P . Hyperinflammatory shock in children during COVID‐19 pandemic. Lancet. 2020;395(10237):1607‐1608. 32386565 10.1016/S0140-6736(20)31094-1 PMC7204765

[51] Verdoni L , Mazza A , Gervasoni A , et al. An outbreak of severe Kawasaki‐like disease at the Italian epicentre of the SARS‐CoV‐2 epidemic: an observational cohort study. Lancet. 2020;395(10239):1771‐1778. 32410760 10.1016/S0140-6736(20)31103-X PMC7220177

[52] Nakra NA , Blumberg DA , Herrera‐Guerra A , Lakshminrusimha S . Multi‐system inflammatory syndrome in children (MIS‐C) following SARS‐CoV‐2 infection: review of clinical presentation, hypothetical pathogenesis, and proposed management. Children (Basel). 2020;7(7):69. 10.3390/children7070069 PMC7401880 32630212

[53] Rowley AH . Understanding SARS‐CoV‐2‐related multisystem inflammatory syndrome in children. Nat Rev Immunol. 2020;20(8):453‐454. 32546853 10.1038/s41577-020-0367-5 PMC7296515

[54] Consiglio CR , Cotugno N , Sardh F , et al. The immunology of multisystem inflammatory syndrome in children with COVID‐19. Cell. 2020;183(4):968‐981. 32966765 10.1016/j.cell.2020.09.016 PMC7474869

[55] Sharma G , Hanania NA , Shim YM . The aging immune system and its relationship to the development of chronic obstructive pulmonary disease. Proc Am Thorac Soc. 2009;6(7):573‐580. 19934352 10.1513/pats.200904-022RM PMC5820858

[56] Meftahi GH , Jangravi Z , Sahraei H , Bahari Z . The possible pathophysiology mechanism of cytokine storm in elderly adults with COVID‐19 infection: the contribution of "inflame‐aging". Inflamm Res. 2020;69(9):825‐839. 32529477 10.1007/s00011-020-01372-8 PMC7289226

[57] Caruso C , Buffa S , Candore G , et al. Mechanisms of immunosenescence. Immun Ageing. 2009;6:10. 19624841 10.1186/1742-4933-6-10 PMC2723084

[58] Nikolich‐Zugich J , Knox KS , Rios CT , Natt B , Bhattacharya D , Fain MJSARS‐CoV‐2 and COVID‐19 in older adults: what we may expect regarding pathogenesis, immune responses, and outcomes. Geroscience. 2020. 10.1007/s11357-020-00193-1 PMC7196177 32363428

[59] Vijay R , Hua X , Meyerholz DK , et al. Critical role of phospholipase A2 group IID in age‐related susceptibility to severe acute respiratory syndrome‐CoV infection. J Exp Med. 2015;212(11):1851‐1868. 26392224 10.1084/jem.20150632 PMC4612096

[60] Karczewski J , Śledzińska E , Baturo A , et al. Obesity and inflammation. Eur Cytokine Netw. 2018;29(3):83‐94. 30547890 10.1684/ecn.2018.0415

[61] de Heredia FP , Gómez‐Martínez S , Marcos A . Obesity, inflammation and the immune system. Proc Nutr Soc. 2012;71(2):332‐338. 22429824 10.1017/S0029665112000092

[62] Engin AB , Engin ED , Engin A . Two important controversial risk factors in SARS‐CoV‐2 infection: obesity and smoking. Environ Toxicol Pharmacol. 2020;78:103411. 32422280 10.1016/j.etap.2020.103411 PMC7227557

[63] Ritter A , Kreis NN . Obesity and COVID‐19: molecular mechanisms linking both pandemics. Int J Mol Sci. 2020;21:16. 10.3390/ijms21165793 PMC7460849 32806722

[64] Xie X , Chen J , Wang X , Zhang F , Liu Y . Age‐ and gender‐related difference of ACE2 expression in rat lung. Life Sci. 2006;78(19):2166‐2171. 16303146 10.1016/j.lfs.2005.09.038 PMC7094566

[65] Wang A , Chiou J , Poirion OB , et al. Single cell multiomic profiling of human lung reveals cell type‐specific and age‐dynamic control of SARS‐CoV2 host genes. Elife. 2020. 10.7554/eLife.62522 PMC7688309 33164753

[66] Li G , He X , Zhang L , et al. Assessing ACE2 expression patterns in lung tissues in the pathogenesis of COVID‐19. J Autoimmun. 2020;112:102463. 32303424 10.1016/j.jaut.2020.102463 PMC7152872

[67] Li Y , Zhou W , Yang L , You R . Physiological and pathological regulation of ACE2, the SARS‐CoV‐2 receptor. Pharmacol Res. 2020;157:104833. 32302706 10.1016/j.phrs.2020.104833 PMC7194807

[68] Jacobs M , Van Eeckhoutte HP , Wijnant SRA . Increased expression of ACE2, the SARS‐CoV‐2 entry receptor, in alveolar and bronchial epithelium of smokers and COPD subjects. medRxiv. 2020;56:2. 10.1183/13993003.02378-2020 PMC7366177 32675207

[69] Leung JM , Yang CX , Tam A , et al. ACE‐2 expression in the small airway epithelia of smokers and COPD patients: implications for COVID‐19. Eur Respir J. 2020;55:5. 10.1183/13993003.00688-2020 PMC7144263 32269089

[70] Radzikowska U , Ding M , Tan G . Distribution of ACE2, CD147, CD26, and other SARS‐CoV‐2 associated molecules in tissues and immune cells in health and in asthma, COPD, obesity, hypertension, and COVID‐19 risk factors. Allergy. 2020;75(11):2829‐2845. 32496587 10.1111/all.14429 PMC7300910

[71] Ziegler CGK , Allon SJ , Nyquist SK , et al. SARS‐CoV‐2 receptor ACE2 is an interferon‐stimulated gene in human airway epithelial cells and is detected in specific cell subsets across tissues. Cell. 2020;181(5):1016‐1035. 32413319 10.1016/j.cell.2020.04.035 PMC7252096

[72] He L , Ding Y , Zhang Q , et al. Expression of elevated levels of pro‐inflammatory cytokines in SARS‐CoV‐infected ACE2+ cells in SARS patients: relation to the acute lung injury and pathogenesis of SARS. J Pathol. 2006;210(3):288‐297. 17031779 10.1002/path.2067 PMC7167655

[73] Gu H , Xie Z , Li T , et al. Angiotensin‐converting enzyme 2 inhibits lung injury induced by respiratory syncytial virus. Sci Rep. 2016;6:19840. 26813885 10.1038/srep19840 PMC4728398

[74] Bao H , Gao F , Xie G , Liu Z . Angiotensin‐converting enzyme 2 inhibits apoptosis of pulmonary endothelial cells during acute lung injury through suppressing miR‐4262. Cell Physiol Biochem. 2015;37(2):759‐767. 26356266 10.1159/000430393

